# Spatial and temporal variations of microbial community in a mixed plug-flow loop reactor fed with dairy manure

**DOI:** 10.1111/1751-7915.12125

**Published:** 2014-04-01

**Authors:** Yueh-Fen Li, Po-Hsu Chen, Zhongtang Yu

**Affiliations:** 1 Environmental Science Graduate Program, The Ohio State UniversityColumbus, OH, USA; 2 Department of Statistics, The Ohio State UniversityColumbus, OH, USA; 3 Department of Animal Sciences, The Ohio State UniversityColumbus, OH, USA

## Abstract

Mixed plug-flow loop reactor (MPFLR) has been widely adopted by the US dairy farms to convert cattle manure to biogas. However, the microbiome in MPFLR digesters remains unexplored. In this study, the microbiome in a MPFLR digester operated on a mega-dairy farm was examined thrice over a 2 month period. Within 23 days of retention time, 55–70% of total manure solid was digested. Except for a few minor volatile fatty acids (VFAs), total VFA concentration and pH remained similar along the course of the digester and over time. Metagenomic analysis showed that although with some temporal variations, the bacterial community was rather stable spatially in the digester. The methanogenic community was also stable both spatially and temporally in the digester. Among methanogens, genus *Methanosaeta* dominated in the digester. Quantitative polymerase chain reaction (qPCR) analysis and metagenomic analysis yielded different relative abundance of individual genera of methanogens, especially for *Methanobacterium*, which was predominant based on qPCR analysis but undetectable by metagenomics. Collectively, the results showed that only small microbial and chemical gradients existed within the digester, and the digestion process occurred similarly throughout the MPFLR digester. The findings of this study may help improve the operation and design of this type of manure digesters.

## Introduction

Anaerobic digestion (AD) is a microbial-mediated process that converts organic compounds into methane and hydrogen containing biogas and has been attractive for both waste treatment and energy production. Over the past several decades, the main use of AD was for wastewater treatment, and the biogas produced was not typically utilized as an energy source. However, the recent need for renewable alternative energy sources has incentivized AD systems to be implemented primarily for production of biogas from various waste streams, including manure produced from concentrated animal feeding operations (CAFOs) and large dairy farm operations (Yu *et al*., 2010). Because of the large quantities of manure produced and the ease of collecting and pump it from dairy barns to digesters, more AD systems are currently being operated on dairy farms than on other farm operations for biogas production in the United States (http://www.epa.gov/agstar/projects/). Further, AD has been the primary method used by dairy farms, particularly small ones, to produce biogas from manure in developing countries such as Costa Rica, China and India (Hobson, 1990; Lansing *et al*., 2008). In the United States, implementation of anaerobic digesters on dairy farms, mostly large mega dairy farms, has been largely promoted by the AgStar program of Environmental Protection Agency (EPA) (http://www.epa.gov/agstar/).

**Fig. 1:**
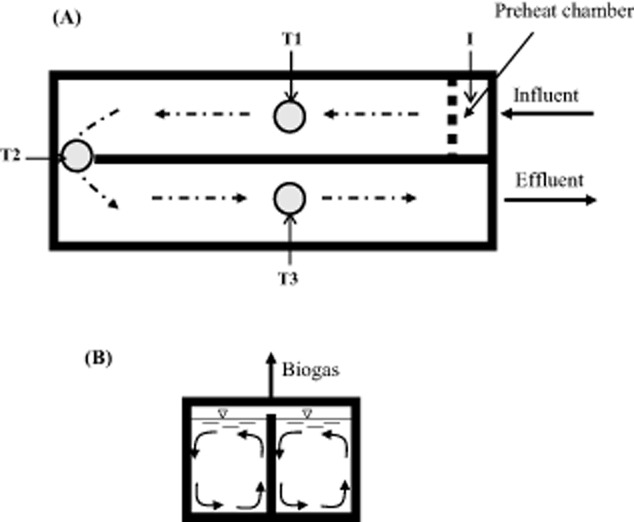
Scheme of a MPFLR.A. Aerial view and sampling locations.B. Cross-section view showing mixing perpendicular to the plug flow. T1, T2 and T3, thermal probe ports through which digester samples were collected.

The mixed plug-flow loop reactor (MPFLR) is an AD system designed and implemented by DVO (Chilton, WI, USA). According to a recent survey conducted in November 2013 by the US Environmental Protection Agency, nearly 40% of the AD systems operated on US dairy farms used the MPFLR design (http://www.epa.gov/agstar/projects/index.html#database). The popularity of MPFLR digesters stem from their simplicity in construction and maintenance along with operational reliability. A MPFLR system is essentially a horizontally oriented U-shaped tank (Fig. 1A). The waste influent enters an MPFLR digester at one end, flows forward and loops back as a ‘plug’, and finally exits from the other end (Yu *et al*., 2010). The digester content is continuously mixed by biogas in the direction perpendicular to the plug-flow of the reactor (Fig. 1B). A portion of the effluent is typically recycled to inoculate the influent and improve AD.

Microorganisms are the driving force for the whole transformation process in anaerobic digesters, and the microbiome therein has been investigated extensively to understand the microbiology underpinning the AD process and to help optimize digester operation, especially in constantly stirred tank reactor (CSTR) and upflow anaerobic sludge blanket (UASB) digesters. However, only a few studies have investigated the microbiome in anaerobic plug-flow digester (Smith and Oerther, 2006; Goberna *et al*., 2009), and no study has been reported that investigated the microbiome (both bacteria and archaea) in any digester that uses the MPFLR design. The objectives of this study were to characterize the microbiome and assess its spatial and temporal variation in a full-scale, mesophilic MPFLR digester operated on a mega-dairy farm in Wisconsin. Results of chemical and metagenomic analyses revealed a relatively stable microbiome in the MPFLR digester that underwent some temporal variations, corroborating the stable operation of this type of anaerobic digester.

## Materials and methods

### Sample collection

The MPFLR digester analysed in the present study is located on a ‘mega'-dairy farm in Wisconsin, USA. This farm has 4000 dairy cows fed a typical corn silage-based total mixed ration (TMR). The digester has been operated stably at 37°C with a hydraulic and solids retention time of 23 days since 2007. The manure slurry is continuously pumped from a manure pit into the digester. The biogas produced is used to power a 1200 kW gas-fired combined heat and power (CHP) system. The influent (I), three locations along the course of the digester (T1, T2 and T3; corresponding to thermal probe locations as shown in Fig. 1A), and the effluent (E) were sampled three times on 3 August (designated as sample S1), 15 September (S2) and 30 September (S3) in 2011. The samples were frozen immediately after collection and shipped to our laboratory overnight where they were stored at −80°C prior to analysis.

### Chemical analysis

Volatile fatty acid (VFA) concentrations of the digester samples were measured using gas chromatography (HP 5890 series, Agilent Technologies, Santa Clara, CA) fitted with a flame ionization detector (FID) and a Chromosorb W AW packed-glass column (Supelco, Sigma-Aldrich, St. Louis, MO, USA) as described previously (Zhou *et al*., 2011). The pH, contents of total solid (TS) and volatile solid (VS) were also measured as described previously (Association of Official Analytical Chemists, 1990).

### Propidium monoazide (PMA) pretreatment and DNA extraction

To exclude DNA from non-viable microorganisms in the samples from being analysed, all the samples were treated with PMA as described previously (Nocker *et al*., 2007) with slight modification. Briefly, 0.5 g of each sludge sample was centrifuged at 10 000 × g at 4°C for 5 min to separate the microbial biomass from the supernatant. The supernatant was aspirated, and the biomass fraction was resuspended in 1 ml of TE buffer. To each microbial biomass sample, 2.5 µl of PMA (20 mM) (Biotium, Hayward, CA) was added to achieve a final concentration at 50 µM. The samples were then incubated in the dark for 5 min at room temperature, followed by light activation by exposure to a 600 Watt halogen light at a distance of 20 cm for 5 min. To avoid excessive heating during the light activation step, the samples were left on ice on a rocker. After PMA treatment, total metagenomic DNA was extracted from each sample using the repeated bead beating plus column purification (RBB + C) method (Yu and Morrison, 2004a). The integrity of the extracted DNA was evaluated by agarose gel (1.0%) electrophoresis, and the concentrations were determined using a NanoDrop 1000 spectrophotometer (Thermo Scientific, Wilmington, DE, USA).

### Polymerase chain reaction (PCR) and denaturing gradient gel electrophoresis (DGGE)

The bacterial and archaeal communities were initially examined by PCR-DGGE using domain-specific primers (Table 1) as described previously (Yu and Morrison, 2004b; Yu *et al*., 2008). Briefly, the V3 region of the 16S rRNA gene was amplified from 100 ng of each metagenomic DNA sample using the primer set GC-357f/519r for bacteria and 344f/GC-519r for archaea. Bovine serum albumin (BSA) was included (670 ng µl^−1^ final concentration) in all PCR reactions to attenuate potential inhibition. A touch-down thermal program (61°C with 0.5°C/cycle decrement for 10 cycles followed by 25 cycles at 56°C for primer annealing) was used to maximize specificity. The PCR was ended with a final extension step at 72°C for 30 min to eliminate the artifactual double DGGE bands created from possible heteroduplex (Janse *et al*., 2004). Size and quality of the PCR products were verified using agarose gel (1.2%) electrophoresis before running DGGE using a PhorU system (Ingeny, Leiden, NL). A total of 15 µl of PCR products was resolved on 8% polyacrylamide gel (37.5:1) with denaturants gradients from 40% to 60% (100% denaturants consisted of 40% (v/v) formamide and 7 M urea). The gels were stained with SYBR Green I (Invitrogen, Carlsbad, CA) and visualized using a Kodak Gel Logic 200 imaging system (Eastman Kodak, Rochester, NY, USA).

**Table 1:** Primers used in the present study

Analysis	Primers/probes	Sequences (5'–3’)	Target	Ta (^0^C)	Amplicon size (bp)	Reference
DGGE	344F	ACGGGYGCAGCAGGCGCGA	Archaea	56	191	Yu and colleagues (2008)
	GC-519R^a^	ATTACCGCGGCKGCTG				
	GC-357F^a^	CCTACGGGAGGCAGCAG	Bacteria	56	194	Yu and Morrison (2004b)
	519R	ATTACCGCGGCKGCTG				
Pyrosequencing	ArcF-A^b^	WCYGGTTGATCCYGCCRG	Archaea	56	534	Nelson (2011)
	ArcR-B^c^	YGGTRTTACCGCGGCGGCT				
	BactF-A^b^	AKRGTTYGATYNTGGCTCAG	Bacteria	56	532	Nelson (2011)
	BactR-B^c^	GTNTBACCGCDGCTGCTG				
qPCR	Mbt-202F	CGCCTAAGGATGGATC	*Methanobacterium*	60	148	
	Mbt-341Taq^d^	FAM-CGCGAAACCTCCGCAATGC-BHQ				Nelson (2011)
	Mbt-399R	TAAGAGTGGCACTTGGGK				
	Mcu-934F	AGGAATTGGCGGGGGAGCAC	*Methanoculleus*	60	309	Franke-Whittle and colleagues (2009); Shigematsu and colleagues (2003)
	Mcu-1023Taq^d^	Cy5-GAATGATTGCCGGGCTGAAGACTC-BHQ				
	Mcu-1200R	CCGGATAATTCGGGGCATGCTG				
	MB1b	CGGTTTGGTCAGTCCTCCGG	*Methanosarcina*	60	271	Shigematsu and colleagues (2003)
	SAR761Taq^d^	HEX-ACCAGAACGGGTTCGACGGTGAGG-BHQ				
	SAR835R	AGACACGGTCGCGCCATGCCT				
	MS1b	CCGGCCGGATAAGTCTCTTGA	*Methanosaeta*	60	272	Shigematsu and colleagues (2003)
	SAE761Taq^d^	FAM-ACCAGAACGGACCTGACGGCAAGG-BHQ				
	SAE835R	GACAACGGTCGCACCGTGGCC				
	Mcp193F	TCCTCGAAAGATCCGTC	*Methanocorpusculaceae*	60	314	Goberna and colleagues (2010)
	Mcp491R	GCCYTGCCCTTTCTTCAC				This study

a. A 40 nt GC clamp was attached at the 5’ end of the primer GC-519R and GC-357F.

b. A 21 nt adaptor A and TCAG key was attached at the 5’ end.

c. A 25 nt adaptor B and TCAG key and a 10 nt sample-specific barcode sequence was attached at the 5’ end.

d. The probes contain a fluorescence dye at 5’ end and a quencher BHQ at 3’.

### 16S rRNA gene pyrosequencing

The V1–V3 hypervariable region (about 500 bp) of the 16S rRNA gene was amplified from each DNA sample using fusion primers ArcF-A and ArcR-B (for archaea) or BactF-A and BactR-B (for bacteria) (Table 1). Both primer sets were modified from domain-specific primers that have been commonly used to amplify the V1–V3 region (Nelson, 2011) by introducing degenerate bases for maximal inclusiveness (Baker *et al*., 2003). One hundred nanogram of each genomic DNA was used in each PCR with the following thermal program: initial denaturation at 95°C for 5 min, followed by 35 cycles of denaturing at 94°C for 30 s, annealing at 54°C for 30 s, and extension at 72°C for 1 min. The PCR cycles were ended with a final extension at 72°C for 10 min. Quality of the PCR products were examined using agarose gel (1.0%) electrophoresis. The bands of the expected size (approximately 550 bp) were excised, and the DNA amplicons were purified using a Qiaquick Gel Extraction Kit (Qiagen, Valencia, CA, USA). The purified amplicon libraries were pooled at equimolar ratio to a final concentration of 20 ng DNA µl^−1^ for both bacteria and archaea, and the two pools were then combined at a 9:1 molar ratio and sequenced using a Roche GS FLX Titanium system at the Plant-Microbe Genomics Facility at The Ohio State University.

### Analysis of sequences

The 16S pyrosequencing reads were processed and analysed using the bioinformatics package QIIME, version 1.7 (Caporaso *et al*., 2010,2010). The sequences for each sample were demultiplexed using the standard options for processing pyrosequencing data with the following modifications (maximum sequence length 600 bp; sliding window test of quality scores: 50 bp; and maximum length of homopolymer run: 8). After demultiplexing, the sequences were denoised using the QIIME Denoiser following the recommended protocol (Reeder and Knight, 2010). The denoised reads were clustered into operational taxonomic units (OTUs) by clustering against the V1–V3 region of the Greengenes 97% reference OTUs (2013-08 release), followed by *de novo* OTU clustering of sequences that were not assigned to a reference OTU. The retained sequences were then aligned with the V1–V3 region of Greengenes core set ‘gg_13_8’ (DeSantis *et al*., 2006) using PyNAST (Caporaso *et al*., 2010,2010). Probable chimeric sequences were identified by Chimera Slayer (Haas *et al*., 2011) and removed. uClust (Edgar, 2010) was used to cluster the sequences into species-level OTUs at 0.03 distance. Lane mask was applied to the representative sequences from each OTU, and an approximately maximum-likelihood tree was constructed using FastTree (Price *et al*., 2010). Each OTU representative sequence was assigned to a taxon using RDP Classifier (Wang *et al*., 2007) against the newly released Greengenes reference OTU builds ‘gg_13_8’ (http://greengenes.secondgenome.com/downloads/database/13_8). Because variation in number of sequences among samples can significantly influence comparative analysis of microbial communities (Gihring *et al*., 2012), rarefaction was performed to normalize the uneven numbers of sequences among samples before diversity analysis. Observed OTU numbers, Shannon and Simpson diversity indices, Shannon equitability, along with the phylogenetic distance were calculated as the indicators for alpha diversity. A weighted distance matrix was generated based on the phylogenetic tree using UniFrac (Lozupone and Knight, 2005), and the beta diversity of bacterial community among the samples was compared using principal coordinate analysis (PCoA) implemented in QIIME. The distribution of major bacterial OTUs was visualized using heatmap generated using the software GAP (Wu *et al*., 2010). Pearson's correlation coefficients were calculated among the samples and among the OTUs to examine the similarity of the profiling. Hierarchical clustering trees were generated using the rank-two ellipse seriation method (Chen, 2002; Wu *et al*., 2010) to sort the samples and OTUs.

### Quantification of methanogens

Quantitative real-time PCR (qPCR) was used to quantify the major genera of methanogens commonly found in anaerobic digesters using genus-specific primers (Table 1). Individual sample-derived qPCR standards were prepared as described previously (Yu *et al*., 2005). Briefly, the genomic DNA extracted from all the samples were pooled together and used as template in PCR reaction using each genus-specific primer set. The PCR was performed as follows: denaturation at 95°C for 5 min, followed by 35 cycles of denaturation at 95°C for 30 s, annealing at the respective temperature for each primer set listed in Table 1 for 30 s, and extension at 72°C for 30 s. The PCR reaction was finished with a final extension at 72°C for 10 min. The PCR amplicons, which were derived from all the members of the target genus rather than a single strain, were verified by agarose gel (1.2%) electrophoresis and then purified using a Qiaquick Gel Extraction Kit (Qiagen, Valencia, CA). The purified amplicon was quantified using a NanoDrop 1000 spectrophotometer (Thermo Scientific, Wilmington, DE) and used as the qPCR standards.

TaqMan-based and SYBR green-based qPCR assays including melting curve analysis were performed on a Mx 3000p real-time PCR system (Stratagene, La Jolla, CA, USA) as described previously (Nelson, 2011; Wang *et al*., 2012) using serial (1:10) dilution (10^2^–10^8^ copies/reaction) of each respective qPCR standard (R^2^ > 0.99 for all standard curves). TaqMan-based qPCR was performed using two-step amplification: initiation at 95°C for 5 min, followed by 40 cycles of 95°C for 15 s and 60°C for 1 min with the fluorescence signal detected at the end of the 60°C primer annealing/extension step. For the SYBR green-based qPCR, the thermal program was set as: initiation at 95°C for 5 min, followed by 40 cycles of 95°C for 30 s, 60°C for 30 s, 72°C for 40 s, and 86°C for 16 s. Dissociation curve was generated using 95°C for 1 min, 60°C for 30 s and 95°C for 30 s. Fluorescence signal was collected at the end of the 72°C and 86°C steps (end point) of the 40 cycles and at the ramping period from 60°C to 95°C (all point) of the dissociation curve step. Baseline and threshold were calculated with Mx 3000p software using the fluorescence acquired at the end of 86°C for 16 s, whereas the primer dimers were completely denatured and would not contribute to the fluorescence (Yu *et al*., 2005). Samples with a C_t_ value below that of the no-template control were considered as below the detection limit.

### Statistical analysis

The quantitative PCR data were first log_10_ transformed to improve normality and then analysed using the GLM Procedure of SAS 9.2 (SAS Institute, Cary, NC, USA). The Newman–Keuls test was used as the post-hoc test, with significant difference declared at *P* value ≤ 0.05.

### Data availability

The QIIME demultiplexed files for each sample are available individually for download from National Center for Biotechnology Information Short Read Archive database under the accession number SRP028717.

## Results

### Chemical characteristics of the samples

The pH values of all the samples were very similar, at about 8.0. The influent samples, except sample S1I, had a much higher TS content than the digester samples (Table 2). Inside the digester, TS content varied along the course of the digester, but not consistently decreased. A similar trend was observed for VS content. The TS and VS contents were much lower in the effluent sample than in the influent samples (except for sample S1I). Removal of TS or VS could not be calculated because of the temporal variations in TS and VS content and unknown recirculation rate of the effluent. No information was available on the yields of biogas or methane. The biogas composition was analysed during the period when the digester samples were collected for this study. The result showed that the biogas contained about 60% methane and 40% CO_2_, and this ratio remained relatively stable over time. The electric output of the CHP system that was fuelled by the biogas from the MPFLR digester ranged from 537 to 605 kWh over the sampling period.

**Table 2:** Concentrations of volatile fatty acid (VFA) and content of total solid (TS) and volatile solid (VS)

Sampling time	Sample location	TS (%)	VS (%)	VFA concentration (mM)						Total VFA (mM)

				Acetic Acid	Propionic acid	Isobutyric acid	Butyric acid	Isovaleric acid	Valeric acid	
8/3/2011	I	3.2	2.4	32.55	7.63	0.49	4.37	2.33	7.65	55.01
	T1	3.3	2.5	2.96	0.60	0.00	0.47	0.27	2.84	7.14
	T2	3.3	2.4	8.47	0.92	0.00	0.34	0.19	1.93	11.85
	T3	2.6	2.0	10.28	0.74	0.00	0.29	0.08	1.34	12.74
	E	2.8	2.1	10.65	0.30	0.00	0.28	0.00	1.26	12.49
9/15/2011	I	7.4	6.1	18.36	9.88	1.00	3.60	1.27	2.52	36.63
	T1	2.9	2.1	9.53	0.53	0.00	0.35	0.10	0.89	11.40
	T2	3.7	2.8	9.12	0.27	0.00	0.24	0.00	0.68	10.31
	T3	3.1	2.2	9.61	1.69	0.00	0.24	0.00	0.64	12.18
	E	3.3	2.4	10.93	0.31	0.00	0.31	0.08	0.76	12.39
9/30/2011	I	13.7	11.6	29.22	5.73	0.31	2.37	0.62	1.09	39.35
	T1	4.1	3.0	8.86	0.84	0.06	0.52	0.11	0.58	10.97
	T2	4.0	2.9	11.87	0.55	0.00	0.36	0.08	0.53	13.38
	T3	2.7	1.8	10.16	0.31	0.00	0.24	0.00	0.47	11.17
	E	3.9	2.8	9.04	0.30	0.00	0.29	0.00	0.39	10.01

Volatile fatty acids are intermediates and important indicators of stability of AD, and thus VFA concentrations in the samples were analysed. The total VFA was relatively higher in all the influent samples (37–55 mM), but it decreased to approximately 7–13 mM in the digester samples and the effluents (Table 2). Acetic acid, the major VFA, had a concentration ranging from 19 to 32 mM in the influents and approximately 10 mM in all the other samples except sample S1T1. The other VFA had much lower concentrations, falling below 1 mM except in the influent samples and valeric acid in samples S1I. Overall, both total VFA and individual VFA did not exhibit a large gradient in concentration along the course of the digester.

### DGGE profiles of bacterial and archaeal communities

Duplicated DGGE analysis consistently showed that except for the influent samples, the DGGE profiles for both archaea and bacteria were very similar along the course of the digester and for the effluents (Supporting Information Fig. S1). For archaea, no significantly difference in DGGE banding patterns was observed among all the samples, except sample S3I that showed different intensity for most of the DGGE bands from the other samples (Supporting Information Fig. S1A). For bacteria, the influent samples had different DGGE banding patterns than the samples collected inside the digester and from the effluents, and the banding patterns were similar among all the samples collected along the course of the digester and the effluents (Supporting Information Fig. S1B). These results suggested that the bacterial and archaeal communities were quite similar along the course of the digester.

### Phylogenetic diversity and composition of archaeal communities

In total, 41 938 quality-checked archaeal 16S rRNA gene sequences were obtained, with an average of 2796 sequences per sample. Collectively, 154 species-level OTUs were found, including 56 singletons. Except eight OTUs that were assigned to the genus *Candidatus Nitrososphaera* in the phylum *Crenarchaeota*, all the remaining 146 OTUs were classified to the phylum *Euryarchaeota*. Most of the *Euryarchaeota* sequences were classified to the class *Methanomicrobia*. Four OTUs that each contained more than 1% of the total archaeal sequences together accounted for 96.3% of the total archaeal sequences. Three of these four abundant OTUs (OTU #549, #224 and #500) were represented by 85.6%, 7.7% and 1.1% of the archaeal sequences, respectively, and were all assigned to the genus *Methanosaeta*, while the other (OTU #201) contained 2.0% of the archaeal sequences, and was assigned to the family *Methanocorpusculaceae*. Genera *Methanoculleus*, *Methanomethylovorans* and *Methanosarcina* were also found, but each was represented by less than 20 sequences. In sample S3I, 99% of the archaeal sequences were clustered into one OTU (OTU #201) that was classified to family *Methanocorpusculaceae*. In the rest of the samples, about 99% of the archaeal sequences were clustered into 98 OTUs classified to genus *Methanosaeta.* No sequence was assigned to genus *Methanobacterium*.

### Phylogenetic diversity and composition of bacterial communities

**Fig. 2:**
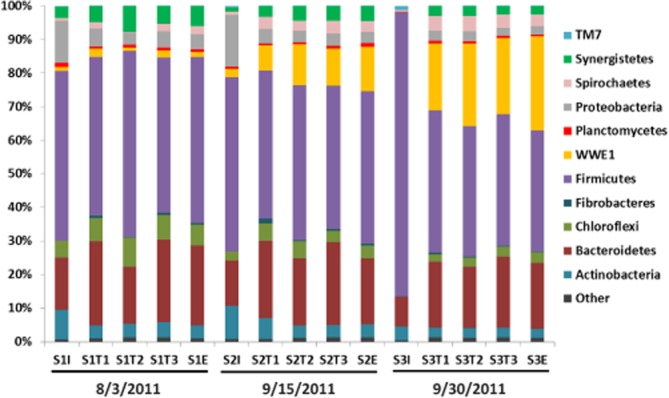
Major bacterial phyla (represented by > 1% total bacterial sequences in at least one sample) in each sample. Sample designations: I, influent; T1, T2 and T3, locations along the course of the digester; E, effluents of the MPFLR digester. Sampling dates were stated under the labels.

Quality-checked bacterial 16S rRNA gene sequences amounted 83 469 in total, with an average of 5564 sequences per sample. These sequences were clustered into 4115 species-level OTUs, of which 2671 were singleton OTUs. Only about 1% (889 sequences) of the total bacterial sequences was classified as unclassified bacteria at phylum level, which as clustered into 484 OTUs with 442 of them being singletons. Collectively, the bacterial community in the samples contained 33 phyla, including candidate phyla BRC1, GN02, NBK19, OD1, OP9, OP11, SR1, TA06, TM6, TM7, WPS-2, WS1 and WWE1. The composition of major bacterial phyla each with a relative abundance greater than 1% in each sample was shown in Fig. 2. These major known phyla accounted for 96.5% of the total bacterial sequences obtained. Consistent with the DGGE result, except for the influents, the bacterial diversity at the phylum level was similar at different locations of the digester and the effluents, with *Firmicutes* (35–54%) and *Bacteroidetes* (13–24%) being the predominant phyla at all the sampling times. In addition, the proportion of the candidate phylum WWE1 notably increased over the sampling period and became one of the predominant phyla in these samples with a relative abundance around 1–2.5% for the first sampling time and 20–27% by the end of the sampling period. 99.8% of the sequences observed in this phylum WWE1 were classified to the candidate genus *Candidatus Cloacamonas*. A lower diversity at the phylum level was observed in the influent samples, especially in sample S3I in which *Firmicutes* alone accounted for 83% of total bacterial sequences, than in the other samples. *Actinobacteria* and *Proteobacteria* were more predominant in the influents than in the digesters except S3I that had no detectable *Proteobacteria*.

Alpha diversity indices in terms of observed OTU numbers, Simpson index, Shannon index, Shannon equitability and phylogenetic distance were calculated to elucidate the diversity of the bacterial community in the samples (Table 3). In general, the bacterial community was found more diverse in the influent samples than in the other samples except that the phylogenetic distance was noticeably lower in sample S3I. Moreover, the community diversity decreased overtime during the sampling period.

**Table 3:** Alpha diversity analysis on the MPFLR samples

Sampling time	Sample location	Observed	Simpson (*D*)	Shannon (*H*)	Shannon's equitability (*E_H_*)	Phylogenetic distance
8/3/2011	I	580	0.99	5.65	0.89	113.45
	T1	486	0.99	5.18	0.84	99.10
	T2	399	0.97	4.70	0.79	84.41
	T3	442	0.99	5.14	0.84	90.03
	E	457	0.99	5.16	0.84	96.92
9/15/2011	I	597	0.99	5.67	0.89	106.73
	T1	476	0.98	5.03	0.82	100.59
	T2	456	0.98	4.87	0.80	95.54
	T3	488	0.98	4.99	0.81	106.67
	E	472	0.98	4.96	0.80	100.65
9/30/2011	I	544	0.99	5.45	0.87	83.47
	T1	492	0.95	4.71	0.76	105.44
	T2	450	0.93	4.45	0.73	93.30
	T3	426	0.94	4.46	0.74	92.08
	E	398	0.91	4.16	0.69	88.46

The goodness of fit test for multinomial distribution with equal probability was used to test if statistically different distribution pattern was present for each of the major taxa with a normalized relative abundance > 20% in at least one sample (the sum of the relative abundance was normalized to 100% for all the samples) and for the corresponding phyla the major taxa belonged to. Although distribution of the two most predominant bacterial phyla (*Firmicutes* and *Bacteroidetes*) and another major phylum *Planctomycetes* was similar (*P* > 0.05) among the samples, different distributions at lower taxonomic ranks were observed (*P* ≤ 0.05), especially in the influent samples (Table 4). In sample S3I, the phylum *Actinobacteria* was mainly composed of the genus *Bifidobacterium* and the family *Coriobacteriaceae*, while in the other samples, that phylum was mainly composed of the order *Actinomycetales.* In addition, sequences classified to *Bifidobacterium*, a beneficial lactic acid-producing genus commonly found in intestinal track, was almost exclusively observed in the three influents: 49% in S3I, 34% in S2I and 6% in S1I, and less than 2% each in the other influent samples. This observation suggested that the environmental condition in the MPFLR digester did not favour bifidobacterial survival. Furthermore, in the phylum *Firmicutes*, the order *Lactobacillales* was mostly represented by sequences from the influent samples S1I and S2I, while the genus *Streptococcus* were mainly found in the S1 samples. However, another order *Clostridiales* was found most predominant in sample S3I, except for the family *Peptostreptococcaceae* that was found most predominant in S1 samples and the genus *Caldicoprobacter* that was found most predominant in S3 samples. In the phylum *Proteobacteria* and the candidate division TM7 and WWE1, the distribution patterns of the phylum level and the lower taxonomic rank were mostly coherent.

**Table 4:** Distribution of the taxa with notable difference in relative abundance (%) among samples*

Taxon	*p*-value	S1I	S1T1	S1T2	S1T3	S1E	S2I	S2T1	S2T2	S2T3	S2E	S3I	S3T1	S3T2	S3T3	S3E
**p__Acidobacteria**	.001	15	5	1	3	5	5	6	11	7	3		12	7	9	11
f__RB40	< .001	**35**	5	2	4	6	5	2	10	10	1		5	4	4	7
**p__Actinobacteria**	.003	14	5	4	7	6	16	6	7	2	2	7	5	6	3	8
f__C111	< .001	12	12	11	3	10	**22**	5	9	1	1		2	2	2	6
f__Coriobacteriaceae	< .001	3		4	2	2	7	2	7		1	**52**	2	8	1	6
f__Microbacteriaceae	< .001	**20**	4	2	2	8	**22**	2	12		2		12	4	2	8
g__Actinotalea	< .001	**20**	8	1	5	8	18	8	4	3	3		5	4	5	7
g__Bifidobacterium	< .001	6			1	1	**34**	2	2			**49**	2	2	1	
g__Propionicimonas	< .001	**23**	10	10	12	14	2	3	8	1			7		1	8
g__Rhodococcus	< .001	8	1	4	4	18	**21**	8	2	4	3	3	6	7	7	5
o__Acidimicrobiales	< .001	**20**	12	4	8	6	12	4	13	1	2		3	3	4	11
**p__Bacteroidetes**	**.365**	6	8	4	9	9	5	6	9	4	3	3	8	9	5	12
f__Bacteroidaceae	< .001	5	4	2	5	3	3	3	5	3	2	1	**21**	15	8	**20**
f__Cytophagaceae	< .001	**22**	8	3	6	8	**23**	6	5	2	1		4	5	2	6
f__Flavobacteriaceae	< .001	8	6	2	5	3	**34**	3	5	3	2		5	10	5	10
f__Rikenellaceae	< .001	6	2			1	17	2	2			**66**		2		
f__S24-7	< .001	11	1	1			14					**73**	1			
f__SB-1	< .001	2	12	4	4	8	4	3	3	2	3		14	13	4	**24**
f__Sphingobacteriaceae	< .001	**25**	3	1	4	6	**44**	1	2	2	2		4	2		3
f__Weeksellaceae	< .001	14	5	3	7	6	**34**	2	3	1	1		3	7	4	9
g__Paludibacter	< .001	4	4		6	6	11	2	6	2		7	**20**	13	9	11
**p__Chloroflexi**	.007	8	10	9	13	11	5	6	10	2	2		4	6	4	9
f__A4b	< .001	**32**	10	5	4	14	12	5	3			1	1	4		8
o__Chloroflexales	< .001	**43**	4	1	1	3	13	6	3	3	4		3	9		4
**p__Fibrobacteres**	< .001	1	12	1	11	10		17	8	3	3		12	11	2	9
g__Fibrobacter	< .001	3	15		4	7	1	13	3	4	1	1	**22**	13	3	6
**p__Firmicutes**	**.321**	7	6	5	7	7	8	4	8	3	2	13	7	8	4	9
f__Aerococcaceae	< .001	**25**	2	3	1	2	**44**	2	1	1	2		4	4	3	4
f__Lachnospiraceae	< .001	11	3	2	4	6	10	2	3	1	1	**40**	5	4	3	5
f __Mogibacteriaceae	< .001	6	5	4	8	7	6	3	8	4	2	**22**	7	7	6	5
f__Peptostreptococcaceae	< .001	7	5	**22**	7	11	5	8	5	3	3	4	6	6	2	7
f__Ruminococcaceae	< .001	7	4	1	4	5	14	2	6	2	2	**37**	5	5	2	4
g__Butyrivibrio	< .001	5	3		3	2	7	2	5	1	5	**44**	8	8	2	5
g__Caldicoprobacter	< .001	1	2	1	4	4	1	4	9	4	2		17	19	9	**22**
g__Dorea	< .001	9	2	1	1	2	13	2	2	1		**61**	1	5	2	
g__Facklamia	< .001	**33**	2	1	2		**51**	2	2	1	1		1	2	1	1
g__Mogibacterium	< .001	2	2		4		10		8		2	**50**	4	8	8	4
g__Oscillospira	< .001	13	5	4	2	2	17	2	2	2	1	**30**	9	4	2	8
g__Proteiniclasticum	< .001	**29**	5		3	8	**27**	3	2	2	2		2	3	7	7
g__RFN20	< .001	5	5	1	8	7	1	5	4	4	3		**23**	11	4	**20**
g__Ruminococcus	< .001	4	1		2	2	15	1	4	1	1	**49**	6	5	3	4
g__Solibacillus	< .001	8	12		2	8	**59**	2	2	4				2	2	
g__Streptococcus	< .001	12	**20**	8	18	15	3		2			1	2	7	3	8
g__Tissierella_Soehngenia	< .001	11	4	7	11	4	7	8	**20**		7		8	1	3	9
g__Trichococcus	< .001	**31**	2		2	2	**48**	2	4	1	1		2	2	1	2
o__Lactobacillales	< .001	**26**	**22**	8	17	17	8				1		1	1		
**p__Planctomycetes**	**.352**	12	6	6	9	7	7	4	6	3	4	2	9	9	6	6
g__Planctomyces	< .001	**24**	3	8	10	1	**20**		7		1		7	7	6	6
**p__Proteobacteria**	< .001	18	7	3	7	7	**22**	4	6	2	2		5	6	2	7
f__Alcaligenaceae	< .001	**24**	7	4	5	9	19	3	3	1	2	1	6	10	4	2
f__Erythrobacteraceae	< .001	18	7	3	7	7	**29**	5	3	2	2		5	5	2	5
f__Hyphomicrobiaceae	< .001	**25**	3	1	7	7	**22**	6	10	2	2		5	4	3	3
f__Phyllobacteriaceae	< .001	14	7	5	9	5	**20**	5	8	2	2		7	7	2	7
f__Sphingomonadaceae	< .001	5	5	2	3	3	**40**	5	12	2	3		10	5		3
f__Xanthomonadaceae	< .001	**23**	4	2	4	6	**32**	2	5	2	1		6	4	1	8
g__Acinetobacter	< .001	18	6			2	**51**	3	6	2	3	2	2	3		3
g__Desulfobulbus	< .001	12	4	**21**	16	3	1	1	4	1	1		10	9	5	12
g__Devosia	< .001	15	7	8	8	8	**20**	7	9	1			5	7		4
g__Hydrogenophaga	< .001	15	3	1	9	7	**21**	1	7	3	2		5	9	4	11
g__Luteimonas	< .001	**22**	8	1	6	8	**26**	5	4	2	1		4	4	3	5
g__Mesorhizobium	< .001	12	6	4	7	4	**22**	4	11	1	1		6	11	1	10
g__Paracoccus	< .001	14	7	3	14	7	**22**	6	5	2	4		4	5	3	6
g__Pseudomonas	< .001	15	4	4	4	11	**23**	1	4	1	3		10	1	6	11
o__Rhizobiales	< .001	**21**	7	5	7	5	16	4	8	2	3		7	6	3	5
**p__Spirochaetes**	< .001	2	4	1	6	7	3	6	9	4	3	1	13	17	7	16
c__Spirochaetes	< .001	4			8	6	2	8	6	4	4	2	**22**	14	8	14
g__Sphaerochaeta	< .001	1	1		2	2	4	5	10	4	4		16	**22**	9	19
**p__TM7**	< .001	6	2	2	4	3	17	1	3	1	1	**45**	4	5	1	4
f__F16	< .001	3	2			2	8	1		1	1	**74**	3	3	1	1
**p__WWE1**	< .001	1	1		1	1	2	3	9	3	3		15	**21**	10	**30**
g__Candidatus Cloacamonas	< .001	1	1		1	1	1	3	9	3	3		15	**21**	10	**30**

* Relative abundance greater than 20% was shown in bold.

**Fig. 3:**
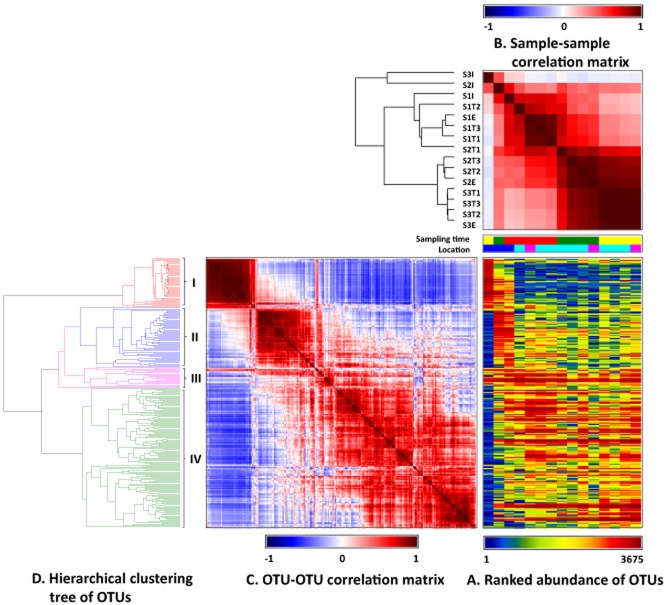
Heatmap of the top 245 OTUs represented by > 50 reads in the MPFLR samples.

The OTUs (245 in total) that were each represented by at least 50 sequences were used to further compare the bacterial communities among the samples. The phylogenetic tree built based on these major OTUs shared similar topology with the tree generated from all the OTUs, suggesting that these ‘major’ OTUs can represent the structures of the bacterial community of all the samples. The occurrence of the OTUs was compared among all the samples using heatmap, sample–sample correlation, OTU–OTU correlation and hierarchical clustering tree of OTUs (Fig. 3). Ranked relative abundance of OTUs in each samples (Fig. 3A) and sample–sample correlation (Fig. 3B) clearly showed that the bacterial community structures of sample S3I and S2I were distinctly different from that of the other samples.

Based on OTU–OTU correlation, the major OTUs were grouped into four groups (Fig. 3C). Group I contained 45 OTUs that were found in the influent samples, especially predominant in S3I, but barely observed in the digester samples or the effluent samples. Forty of these OTUs were assigned to the order *Clostridiales*, with 17 and 15 further assigned to the families *Ruminococcaceae* and *Lachnospiraceae* respectively. Of the remaining five OTUs, one was assigned to the candidate division TM7, one was assigned to genus *Bifidobacterium*, and the remaining three were assigned to the order *Bacteroidales*. Group II were composed of 54 OTUs that were mostly observed in S1I and S2I, with few were seen in the other samples. This group of OTUs were mainly classified to the orders *Clostridiales*, *Actinomycetales*, *Lactobacillales* and phylum *Proteobacteria*. Group III contained 19 ubiquitous OTUs that were observed in all the samples. The largest OTU #3 (represented by 2683 sequences) was assigned to the candidate genus SMB53 of *Clostridiaceae*, followed by OTUs #13 (961 sequences) and #14 (834 sequences), both of which were assigned to the family *Peptostreptococcaceae*. Another two abundant OTUs #24 and #25 (both were represented by about 520 sequences) were assigned to the order *Clostridiales* and the genus *Turicibacter* of the class *Bacilli* respectively. Group IV were composed of 127 OTUs that were observed in all samples except S3I. The largest OTU was represented by 8719 sequences and was assigned to the candidate genus *Candidatus Cloacamonas* of the candidate phylum WWE1. This OTU was found mostly in all the digester and effluent samples, especially the samples collected on 30 September 2011. The other OTUs of group IV were assigned mostly to *Bacteroidales* (including three abundant OTUs that were further classified as *Ruminofilibacter xylanolyticum* and seven OTUs classified into the family *Porphyromonadaceae*), *Anaerolineaceae* in the phylum *Chloroflexi*, *Clostridiales* (including two abundant OTUs further classified as *Caldicoprobacter* and six OTUs classified as *Clostridium*), *Ruminococcaceae*, *Erysipelotrichaceae* and *Sphaerochaeta* in the phylum *Spirochaetes*.

### PCoA

**Fig. 4:**
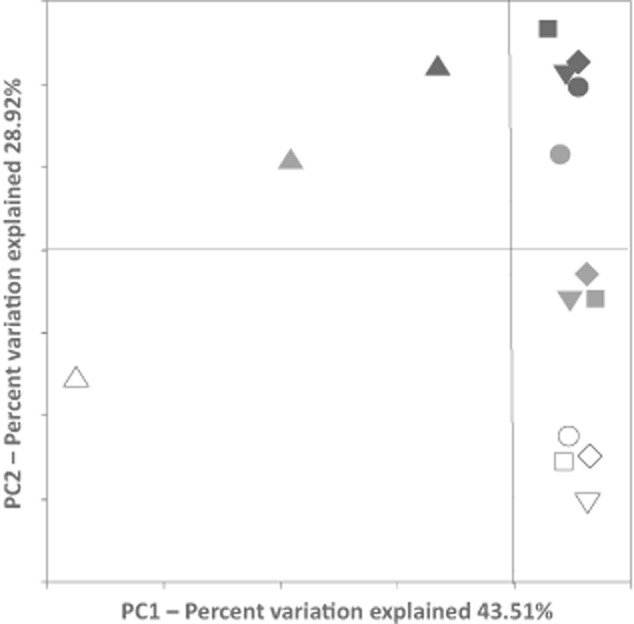
PCoA plot of the bacterial community in the MPFLR samples based on the pyrosequencing analysis. Samples were collected on 3 August (black), 1 September (grey) and 30 September (open) in 2011. Triangles, influents (I); circles, location T1 of the digester; squares, location T2; and diamonds, location T3; inverted triangles, effluents (E).

The diversity of bacterial communities was compared using PCoA on the sequence data (Fig. 4). Collectively, PC1 and PC2 explained 72.4% of the total variation. All the influent samples were separately spotted on the PCoA plot without grouping with each other or with any of the other samples along either PC1 or PC2. Samples collected on the same day grouped together, and the three sampling dates were separated both along PC1, which explained 43.9% of the variation, and along PC2, which explained 25.3% of the variation. Sample S2T1 separated itself from the other S2 samples but stayed close to sample S1 on the PCoA plot. The results showed different bacterial communities in the influents than in the digester samples, and smaller spatial than temporal variations in bacterial community in the MPFLR digester.

### Abundance of major methanogens

**Fig. 5:**
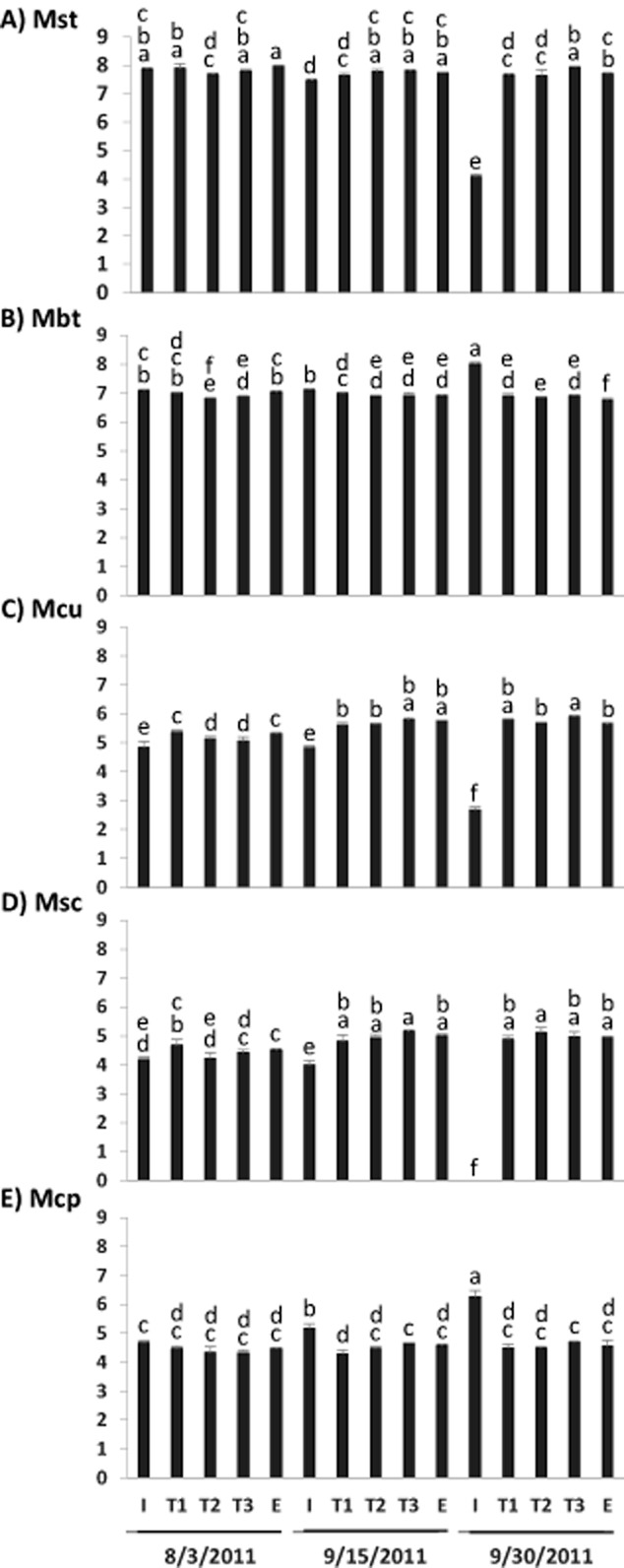
Quantification of five commonly observed genera of methanogens in AD using qPCR.A. Mst, *Methanosaeta*.B. Mbt, *Methanobacterium*.C. Mcu, *Methanoculleus*.D. Msc, *Mehtanosarcina*.E. Mcp, *Methanocorpusculaceae*. Different letters designate significant difference (*P* < 0.05). Labels of the *X*-axis were the same as stated in Fig. 2.

Among the five methanogen genera quantified by qPCR, *Methanosaeta* was the most abundant in all the samples, reaching approximately 8 × 10^7^ copies of 16S rRNA gene per µg metagenomic DNA, except in sample S3I, in which genus *Methanobacterium* dominated with an abundance of 10^8^ copies of 16S rRNA gene per µg DNA (Fig. 5A and B). *Methanobacterium*, which was not detected by the pyrosequencing (see the pyrosequencing results), was the second most abundant genus in most of the samples, reaching at least 10^7^ copies of 16S rRNA gene per µg DNA in all the samples (Fig. 5B). *Methanoculleus* was found at about 10^5^ copies of 16S rRNA gene per µg DNA in all the samples except sample S3I (Fig. 5C). Both the genera *Methanosarcina* and *Methanocorpusculaceae* were detected at about 10^5^ 16S rRNA gene copies µg^−1^ DNA in all the samples, with the exception in sample S3I, where *Methanosarcina* was not detected (Fig. 5D and E). Compared with the other samples, sample S3I had more *Methanobacterium* and *Methanocorpusculaceae* but less *Methanosaeta* and *Methanoculleus*.

## Discussion

Anaerobic digestion is the most suitable technology to manage livestock manure, especially dairy cattle manure, to reduce greenhouse gas emission, harness bioenergy and decrease pathogens. MPFLR digester is the most common technology implemented on dairy farms in the United States. Unlike in CSTR digesters in which the conditions are homogeneous, the plug-flow operation creates a gradient of conditions along the course of MPFLR digesters. To our knowledge, this is the first study that investigated both the archaeal and the bacterial composition inside a MPFLR. To minimize ‘contamination’ with dead microbes from preceding locations of the MPFLR digester, all the samples were subjected to PMA pretreatment, which has shown effective in excluding DNA from non-viable cells from PCR-based analysis. In the present study, DGGE profiling of both archaeal and bacterial communities was used to assess the effect of PMA pretreatment, but no obvious change in DGGE patterns was noted (data not shown). This observation is consistent with the results reported previously that PMA treatment did not significantly affect DGGE patterns of environmental samples (Nocker *et al*., 2007). Using pyrosequencing of 16S rRNA gene amplicons, a later research reported that PMA pretreatment changed the relative abundance of certain bacterial groups in environmental water samples (Nocker *et al*., 2010). Thus, dead microbes were probably also excluded from being analysed in the present study. It is uncertain, however, if all the dead microbes were excluded because the TS content of the manure samples analysed in the present study was greater than the effective range (TS content under 2000 mg l^−1^) of PMA treatment of water samples (Taskin *et al*., 2011) and the dark colour of the manure samples. It remains a technical challenge to be certain to completely exclude dead microbes from being analysed when coloured samples with high TS are analysed using DNA-based techniques.

The reverse primer for the genus *Methanocorpusculum* published previously (Goberna *et al*., 2010) can anneal to the 16S rRNA genes of the four type strains in this genus but not most of the *Methanocorpusculum*-like sequences found in the present study. Therefore, the reverse primer was modified based on the *Methanocorpusculum* sequences archived in the RDP database and the *Methanocorpusculum*-like sequences obtained in the present study. When paired with the forward primer Mcp193F published by Goberna and colleagues (2010), the new reverse primer allowed specific but inclusive amplification of 16S rRNA genes of the genus *Methanocorpusculum* and of bacteria related to *Methanocorpusculum* of the family *Methanocorpusculaceae*, as verified by cloning and sequencing of amplicons produced from this primer pair (data not shown). This primer pair may be used to quantify *Methanocorpusculum* and related bacteria in the family *Methanocorpusculaceae* in anaerobic digesters.

In the present study, *Methanosaeta* was found to be the dominant genus in all the samples except sample S3I, which was dominated by other methanogens (*Methanocorpusculum* according to the pyrosequencing analysis and *Methanobacterium* according to the qPCR analysis). As an acetoclastic methanogen genus with greater affinity for its substrate than other methanogens (Yu *et al*., 2010), the dominance of *Methanosaeta* is consistent with the low acetate concentration found in all the digester samples. The low acetate concentration also explained the low abundance of *Methanosarcina*. This explanation is corroborated by the predominance of *Methanosarcina* in digesters with high acetate concentration (Zinder, 1993; De Vrieze *et al*., 2012), including in a plug-flow digester fed a mixture of crop and liquid cow manure where acetate was at high concentrations (Lerm *et al*., 2012). In a recent study (St-Pierre and Wright, 2013), *Methanosarcina* was found to be the dominant methanogens (> 98% of total methanogen sequences) in two MPFLR digesters implemented on dairy farms in Vermont that co-digest dairy manure and waste from ice cream factory or cheese whey. Although acetate concentrations were not reported in that study, the feeding of readily digestible ice cream waste and cheese whey probably increased the acetate concentrations in those two MPFLR digesters. Nevertheless, methanogen compositions varied considerably in digesters with different designs and feedstocks (Nettmann *et al*., 2010; Zhu *et al*., 2011; St-Pierre and Wright, 2013). Investigation into the relationship between population dynamics of methanogens and physiochemical data of environmental factors will help further understand the performance and stability of anaerobic digesters.

Although the feedstock of the MPFLR digester was from the same dairy farm, the three influent samples had different archaeal populations, with samples S1I and S2I containing *Methanosaeta* as the major methanogen genus while sample S3I *Methanocorpusculaceae* (based on pyrosequencing results) or *Methanobacterium* containing (based on the qPCR result) as the major methanogens. An *in silico* analysis of the archaeal primers used in the present study and the 16S rRNA genes sequences of the genus *Methanobacterium* reveal no mismatch between the primers and their annealing sites (data not shown). Thus, the absence of *Methanobacterium* sequences in the pyrosequencing data might be attributed to the internal sequence of the 16S rRNA genes of this genus, which interfered in amplification during amplicon library preparation and/or during emulsion PCR and/or blocked the pyrosequencing. It should be noted that inability to detect one group changes the relative abundance of all the other groups, screwing the entire community structure. Unlike samples S1I and S2I, which were collected after cow manure was mixed with the recycled effluents of the MPFLR digester, sample S3I was collected, unintendedly, from the manure pits before mixing with the recycled digester effluent. A recent study has shown that *Methanocorpusculum* was predominant in cow manure (Yamamoto *et al*., 2011). Thus, the predominance of *Methanosaeta* in samples S1I and S2I might be attributed to the effluent that was recycled back to the digester, whereas the predominance of *Methanobacterium* and related methanogens in sample S3I was probably explained by the lack of recycled effluent from the digester.

Compared with the distinct bacterial community found in sample S3I, the bacterial community in the two influent samples S1I and S2I was more similar to that found in the digesters samples. As mentioned above, this could also be explained by the recycled portion of the digester effluents. The OTUs of bacteria found mainly in sample S3I were classified to the families *Ruminococcaceae* and *Lachnospiraceae*, and the genus *Bifidobacterium*. These OTUs were likely present in the cow manure. Some of the OTUs (e.g. OTUs #1131 and #1836) were found much more abundant in the influents than in the digester samples. These OTUs were assigned to the families *Ruminococcaceae* and *Lachnospiraceae* and have also been found predominant in bovine faeces (Dowd *et al*., 2008; Rudi *et al*., 2012), suggesting that they were probably faecal bacteria, and they could not proliferate in the digester. The family *Peptostreptococcaceae* contains several known fermenting bacteria in the genera *Anaerosphaera*, *Peptostreptococcus* and *Sporacetigenium* that have been isolated from anaerobic digesters and swine manure (Chen *et al*., 2006; Peu *et al*., 2006; Ueki *et al*., 2009). Members of *Sporacetigenium* can produce acetate and ethanol as main end products, and the type strain of *Sporacetigenium mesophilum* was isolated from an anaerobic digester (Chen *et al*., 2006). The ubiquitous occurrence of OTU #3 (belonging to candidate genus SMB53 of *Clostridiaceae*) and OTU #24 and #25 (belonging to *Peptostreptococcaceae*) might reflect their fitness in the MPFLR digester and potentially important contribution to acidogenesis therein. The gradual overtime increase in relative abundance of the OTUs classified to the candidate genus *C. Cloacamonas* of the candidate division WWE1 implied this genus might play an important role in the MPFLR system. Sequences of bacteria assigned to candidate division WWE1 were first discovered from an anaerobic digester fed municipal sludge (Chouari *et al*., 2005). The genome of a bacterium in this candidate genus, *C. Cloacamonas acidaminovorans*, which was reconstructed via metagenomic sequencing, contained genes likely involved in degradation of protein and amino acid and syntrophic oxidation of propionate (Pelletier *et al*., 2008). Given the high prevalence and relative abundance observed in the present study, bacteria belonging to this candidate genus may be important to the digestion process of dairy manure in MPFLR digesters.

The total retention time inside of the MPFLR digester was 23 days, and the three sampling locations (T1, T2 and T3) were separated in retention time by at least 5 days. Despite temporal variations in both the archaeal and the bacterial communities among the three sampling times, the spatial variations in either community were limited. These limited variation in microbial communities concurred with the similarity in the chemical parameters (e.g. pH, TS, VS and VFA) at these three locations. It was hypothesized that the rather similar chemical conditions and microbial communities along the course of the MPFLR digester was due to two primary reasons. First, dairy manure contains little readily digestible substances (e.g. starch or pectin, which have been digested and absorbed by the cows) but large amounts of faecal microbial cells and undigested recalcitrant cellulose, hemicellulose and lignin. As a result, the digestion processes, particularly hydrolysis, occur slowly as digester content moves through the digester. Second, the MPFLR digesters are not separated into multiple compartments, and the biogas-driven mixing may also create longitudinal mixing as the digester content slowly flows through the digester. Because this was the first bacterial study on any MPFLR digester, there was no study for comparison. However, the above premises seems to be supported by the study of Roy and colleagues (2009) who observed distinctly different archaeal and bacterial communities in a plug-flow digester that was physically divided into multiple compartments and fed with swine manure, which typically contains little recalcitrant cellulose or lignin. Furthermore, it was reported that a gradient of methanogenic activity at different locations was observed in a laboratory-scale plug-flow digester fed with pineapple pulp and peel, which contain readily biodegradable carbohydrates and proteins (Namsree *et al*., 2012). Future studies using tracers and microbial activity-based analysis on samples collected along the course of the MPFLR digesters can help test the above hypotheses. Collectively, the results of the present study provided chemical and microbial evidence for the stable operation of MPFLR digesters. The results also suggest that further increase in hydraulic retention time may further increase biogas yield per unit of dairy manure fed.

## Supporting information

**Fig. S1.** DGGE profiles of archaeal (A) and bacterial (B) communities.

**Table S1.** Distribution and taxonomy assignment of corresponding OTUs in Fig. 4.
