# Supplementary figures and images for: Spatial and temporal variations of microbial community in a mixed plug-flow loop reactor fed with dairy manure
Source: Microb Biotechnol. 2014 Apr 1;7(4):332–46. doi: 10.1111/1751-7915.12125 (PMC4241726; doi:10.1111/1751-7915.12125)

**A) Archaea**

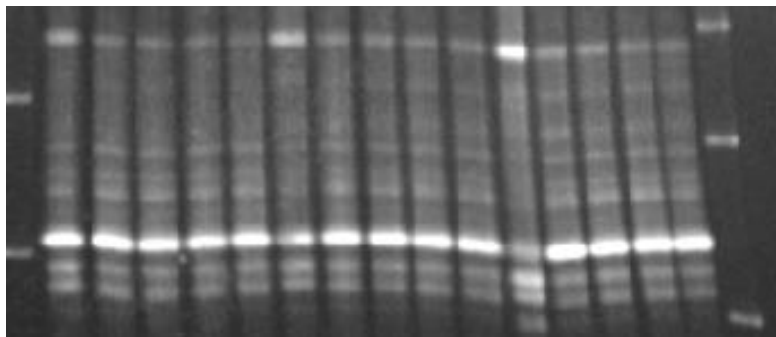

**B) Bacteria**

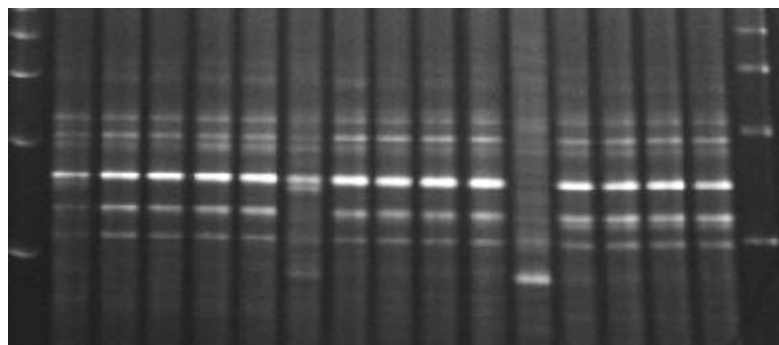

|          |    |    |    |   |           |    |    |    |   |           |    |    |    |   |
|----------|----|----|----|---|-----------|----|----|----|---|-----------|----|----|----|---|
| I        | T1 | T2 | T3 | E | I         | T1 | T2 | T3 | E | I         | T1 | T2 | T3 | E |
| <hr/>    |    |    |    |   | <hr/>     |    |    |    |   | <hr/>     |    |    |    |   |
| 8/3/2011 |    |    |    |   | 9/15/2011 |    |    |    |   | 9/30/2011 |    |    |    |   |

Supplement: Supplementary file 1 [file mbt20007-0332-sd1.pdf]
